# Weight estimation for children aged 6 to 59 months in limited-resource settings: A proposal for a tape using height and mid-upper arm circumference

**DOI:** 10.1371/journal.pone.0197769

**Published:** 2018-06-07

**Authors:** Mark E. Ralston, Mark A. Myatt

**Affiliations:** 1 Department of Pediatrics, Uniformed Services University of the Health Sciences, Bethesda, Maryland, United States of America; 2 Consultant Epidemiologist, Brixton Health, Llawryglyn, Powys, Wales, United Kingdom; TNO, NETHERLANDS

## Abstract

**Importance:**

A simple, reliable tool for rapid estimation of weight in children would be useful in limited-resource settings where current weight estimation tools are not reliable, nearly all global under-five mortality occurs, severe acute malnutrition is a significant contributor in approximately one-third of under-five mortality, and a weight scale may not be immediately available to healthcare professionals including first-response providers.

**Objective:**

To test the accuracy and precision of an existing weight estimation tool based on patient height and mid-upper arm circumference (MUAC) in children between six months and five years of age in low-to-middle income countries.

**Design:**

Data were collected in 2,434 nutritional surveys during 1992–2017 using a modified Expanded Program of Immunization two-stage cluster design.

**Setting:**

Locations in 51 low-to-middle income countries with high prevalence of acute and chronic malnutrition.

**Participants:**

Of 1,848,979 children enrolled in the surveys, a total of 1,800,322 children met inclusion criteria (age 6–59 months; weight ≤ 25 kg; MUAC 80–200 mm) and exclusion criterion (bilateral pitting edema and biologically implausible measurements based on WHO flagging criteria).

**Exposures:**

Weight was estimated by a regression procedure using database height and MUAC.

**Main outcomes and measures:**

Mean percentage difference between true and estimated weight (MPD), proportion of estimates accurate to within ± 10% and ± 20% of true weight (PW10 and PW20), weighted Kappa statistic, and Bland-Altman bias (bias) were reported as measures of tool accuracy. Standard deviation (SD) of the MPD and Bland-Altman 95% limits of agreement (LOA) were reported as measures of tool precision.

**Results:**

The height model fitted for MUAC classes was accurate and precise. MPD was +0.67% (SD = 9.95%); PW10/PW20 were 68.31% (95% CI 68.24%, 68.38%)/94.73% (95% CI 94.69%, 94.76%); and bias (LOA) were +0.06 kg (-1.97 kg; +2.10 kg). For MUAC < 115 mm, PW10/PW20 were 63.91% (95% CI 63.42%, 64.40%)/90.72% (95% CI 90.42%, 91.01%); and bias (LOA) were +0.14 kg (-1.29 kg; +1.56 kg). For 115 mm ≤ MUAC < 125 mm, PW10/PW20 were 76.27% (95% CI 76.03%, 76.51%)/96.36% (95% CI 96.25%, 96.46%); and bias (LOA) were +0.06 kg (-1.20 kg; +1.33 kg). For MUAC > 125 mm, PW10/PW20 were 69.93% (95% CI 69.86%, 70.00%)/95.27% (95% CI 95.24%, 95.30%); and bias (LOA) were +0.05 kg (-2.04 kg; +2.13 kg).

**Conclusions and relevance:**

An updated model estimating weight from height and MUAC in a large database of children aged 6 to 59 months across a wide range of low-to-middle income countries with high prevalence of acute and chronic malnutrition was confirmed to be accurate and precise. A height-based weight estimation tape stratified according to MUAC classes is proposed for children aged 6–59 months in limited-resource settings.

## Introduction

An anthropometric tool for estimation of weight in children would be useful in limited-resource settings where nearly all under-five mortality occurs yet a weight scale may not be immediately available to healthcare professionals including first-response providers [1, 2]. The ideal tool should be simple as well as validated in low-to-middle income countries. Furthermore, as severe acute malnutrition is a significant contributor in approximately one-third of under-five mortality, a tool which accurately estimates total (or actual) body weight instead of ideal body weight would be preferable in order to avoid overestimation of weight in the undernourished child [3]. Two-dimensional weight estimation methods (i.e. based on patient length with adjustment for body habitus) have been found to be more accurate than one-dimensional methods (i.e. based on either length or habitus alone) in predicting total body weight [4, 5].

A weight estimation tool developed from a nutritional survey database of 453,990 children aged 6 to 59 months of age in low-to-middle income countries during 1992–2006 and based on both length and MUAC was found to be more accurate and more precise than existing weight estimation methods (i.e. length-based Broselow Tape and MUAC-based Hong Kong formula) [6]. This study recalibrated and tested the accuracy and precision of this tool with a fourfold increase in nutritional survey data during 1992–2017 across a wider range of low-to-middle income countries.

## Materials and methods

### Surveys

This was a retrospective observational study. Data were collected in 2,434 nutritional anthropometric surveys in 51 low-to-middle income countries over a 25-year period, August 1992 to May 2017. Surveys were performed in locations with high prevalence of both acute and chronic malnutrition due to war, prolonged civil unrest, poor public health environment, and poor food security.

### Data collection and management

The data collection methodology was consistent across the 2,434 surveys. Survey agencies used a “30-by-30” method until 2006 when it was replaced by SMART methodology (which places greater emphasis on standardization and data quality) with one exception: United Nations High Commissioner for Refugees used SENS methodology, which for anthropometry is identical to SMART [7]. A modified Expanded Program of Immunization two-stage cluster survey design was used. Primary sampling units or “clusters” were selected from exhaustive lists of potential primary sampling units (e.g. villages, townships, census enumeration areas) using population proportional sampling. A minimum of *m* = 30 primary sampling units were always selected. The mean overall survey sample size was *n* = 811 children meeting study eligibility criteria. Samples within primary sampling units were taken using the Expanded Program of Immunization proximity sampling method. A single household was selected at random and subsequent households were selected by their proximity to the first household. All eligible children (i.e. children aged 6 to 59 months inclusive) in sampled households were measured. Sampling within each cluster stopped when a fixed sample size (usually *n* ≈ 30) had been met or exceeded.

Weight, height, and MUAC measurements were subject to standardization using the method of Habicht [8]. Single measurements were used and performed always by teams of three people. Children were weighed in minimal clothing (i.e. usually vests / underpants) without shoes using 25 kilogram × 100 gram hanging scales (Salter-Brecknell 235-6S series or similar). Standard pediatric height boards were used. Standing height was recorded in children with a standing height of ≥ 85 cm. Supine length was measured and recorded in children with a standing height < 85 cm. The term “height” is used in this report to refer to both standing height and supine length. Measurement of MUAC was performed at the mid-point of the left arm according to WHO guidelines using non-elastic tapes. In 2009, the WHO diagnostic indicator for severe acute malnutrition defined by MUAC was changed from < 110 mm to < 115 mm [9]. Most surveys will have used UNICEF supply code S0145620 (for surveys undertaken after mid-2009), “MUAC, Child 11.5, Red/PAC-50” or S0145600 "MUAC, Child 11.0 Red/PAC-50" (for surveys undertaken before mid-2009), or similar designs (i.e. copies of the UNICEF design made by / for individual non-governmental organizations) but some will have used TALC design tapes (A/ITC115 "MUAC 115 mm Small Coloured Insertion Tape" or similar).

No clinical data were used. These were not medical experiments involving human subjects and, as such, are exempt from the terms of the Declaration of Helsinki. Whenever possible, data were collected following ethical approval from locally responsible ethics committees. Some data were collected during complex emergencies when no locally responsible ethics committees were operating. In these cases ethical approval was granted solely by the institutional review bodies of the non-governmental organization or United Nations organization which collected the data. Permissions were sought and given by local ministries of health and, where appropriate, by local police departments and military / paramilitary commanders. Identifying data were collected for programmatic purposes (i.e. for recruitment of cases of acute malnutrition into appropriate therapeutic feeding programs) but this data was either not entered or removed prior to data being made available for this analysis. Participation in the surveys was voluntary. In all surveys, the consent procedure was approved. Children were not (and could not be) measured without the consent of their parents or guardians. Verbal informed consent was sought from the primary caregiver of the child. Written consent is almost never sought in these types of survey: it is usually not required; and levels of literacy are often low. The existence of the data is proof of consent.

Data from these surveys were concatenated and the following inclusion criteria applied: age between 6 months and 59 months (inclusive); weight ≤ 25 kg; and MUAC between 80 mm and 200 mm (inclusive). Edema was recorded in all surveys. Children with bilateral pitting edema were excluded because the weight of retained fluid tends to mask what would otherwise be low weight [10]. It should be noted that edema is not well recognized in many clinical and survey contexts. It is likely, therefore, that edema exclusions were limited to grade ++ and grade +++ edema. Children with biologically implausible weight-for-height z-score (WHZ), weight-for-age z-score (WAZ), and height-for-age z-score (HAZ) values (i.e. WHO flagging criteria) were also excluded according to WHO Child Growth Standards guidelines [11].

Of 1,848,979 children in all survey datasets, a total of 1,800,322 children passed the study inclusion and exclusion criteria. Numbers of surveys by country, year, and agency as well as subject demographics, subject anthropometry, and number of children by country are listed in *Table 1.*

**Table 1:** Survey, demographic, and anthropometric characteristics of the study population (n = 1,800,322).

Datasets*	Surveys n*		2,434					
	**Countries n**		51					
	**Countries** (surveys n)**		Afghanistan (43), Albania (1), Angola (22), Bangladesh (28), Benin (7), Burkina Faso (55), Burundi (25), Cameroon (10), Central African Republic (58), Chad (243), Democratic Republic of the Congo (Kinshasa) (266), Côte d'Ivoire (49), Djibouti (14), Eritrea (4), Ethiopia (273), Gambia (8), Guatemala (2), Guinea (12), Guinea Bissau (13), Haiti (49), India (8), Indonesia (3), Jordan (4), Kenya (132), Liberia (55), Madagascar (4), Malawi (16), Mali (14), Mauritania (57), Mozambique (13), Myanmar (22), Nepal (15), Niger (38), Nigeria (107), Pakistan (18), Philippines (12), Rwanda (26), Senegal (7), Sierra Leone (58), Somalia (227), South Sudan (140), Sri Lanka (3), Sudan (144), Tajikistan (5), Thailand (2), Togo (18), Uganda (84), United Republic of Tanzania (8), Yemen (5), Zambia (6), Zimbabwe (1)					
	**Years** **(surveys n)**		1992 (3), 1993 (15), 1994 (35), 1995 (39), 1996 (27), 1997 (33), 1998 (21), 1999 (26), 2000 (39), 2001 (41), 2002 (55), 2003 (54), 2004 (76), 2005 (99), 2006 (70), 2007 (83), 2008 (143), 2009 (155) 2010 (201), 2011 (273), 2012 (261), 2013 (250) 2014 (340), 2015 (79), 2017 (8), Unknown (7)					
	**Agencies** **(surveys n)**		ACF (802), CONCERN (108), FSNAU (207), GOAL (141), IMC (15), IRC (3), MSF (58), PLAN (2), SC (58), TDH (7), UNHCR (355), UNICEF (622), World Vision (18), Zerca y Lejos (1)					
Children***	**Children n**		1,800,322					
	**Sex**	**Males** **n (%)**	910,780 (50.59)					
		**Females n (%)**	889,542 (49.41)					
			**Minimum**	**1**^**st**^ **Quartile**	**Median**	**Mean (SD)**	**3**^**rd**^ **Quartile**	**Maximum**
	**Age (mo)**		6	18	30	31.27 (15.21)	44	59
	**Weight (kg)**		3.3	9.0	11.1	11.20 (2.82)	13.3	25.0
	**Height (cm)**		52.3	76.2	85.1	85.38 (11.62)	94.3	120
	**MUAC (mm)**		80	133	142	142.07 (13.15)	151	179
	**Countries** (children n)*		Afghanistan (47,812), Albania (892), Angola (17,191), Bangladesh (14,554), Benin (7,841), Burkina Faso (41,467), Burundi (14,604), Cameroon (8,530), Central African Republic (36,161), Chad (145,506), Democratic Republic of the Congo (Kinshasa) (226,767), Côte d'Ivoire (23,990), Djibouti (5,257), Eritrea (2,281), Ethiopia (170,704), Gambia (6,721), Guatemala (608), Guinea (9,487), Guinea Bissau (7,131), Haiti (39,465), India (5,145), Indonesia (1,735), Jordan (1,517), Kenya (86,018), Liberia (32,686), Madagascar (3,156), Malawi (15,998), Mali (10,901), Mauritania (36,617), Mozambique (4,417), Myanmar (14,322), Nepal (8,844), Niger (48,995), Nigeria (65,737), Pakistan (14,098), Philippines (6,095), Rwanda (15,559), Senegal (8,421), Sierra Leone (62,913), Somalia (234,981), South Sudan (96,225), Sri Lanka (2,573), Sudan (114,112), Tajikistan (4,297), Thailand (1,795), Togo (11,835), Uganda (54,236), United Republic of Tanzania (5,290), Yemen (1,781), Zambia (2,364), Zimbabwe (690)					

^*^ Numbers given do not include duplicate datasets.

^**^ Surveys were from emergency and refugees settings. The specified country of origin may not reflect nationality or ethnicity of survey respondents.

^***^ Numbers given are for records remaining after the censoring of records with biologically implausible values using the WHO flagging criteria.

**Survey Agencies**: Action Contre La Faim (ACF); CONCERN Worldwide (CONCERN); Food Security and Nutrition Analysis Unit (FSNAU); GOAL; International Medical Corps (IMC); International Rescue Committee (IRC); Médicins Sans Frontières (MSF); Plan International (PLAN); Save the Children (SC); Terre des hommes (TDH); United Nations High Commissioner for Refugees (UNHCR); United Nations Children’s Fund (UNICEF); World Vision; and Zercay Lejos.

### Data management and analysis

Data management and data analyses were performed using the R Language for Data Analysis and Graphics [12]. Weight was estimated using Broselow Tape (BT) 2007 [B] and BT 2011 [A] to the nearest of the 26 BT weight classes (3–36 kg) using measured height in the database [13]. Weight was estimated from database MUAC and height / length using an “un-rotated” linear model initially fitted using a robust regression procedure but then “rotated” using a second linear model [6, 14]. The estimation formula of the second “corrected” linear model was:

$$estimated\;weight=mean-\;\frac{mean-(\alpha_{1}+\;\beta_{1}x)}{\beta_{2}}$$

where:

*mean* The mean estimated weight from the initial “un-rotated” linear model:
  $$estimated\;weight=\alpha_{1}+\beta_{1}x$$
*x* The variable (i.e. MUAC or height) from which weight is estimated.

The corrected model was fitted using the complete dataset (*n* = 1,800,322 children) and yielded weight estimation formulae for both one-dimensional (MUAC only; height only) and two-dimensional (height and MUAC) models. For the two-dimensional method, the height model was fitted separately for three MUAC classes: MUAC < 115 mm corresponding to severe acute malnutrition; 115 ≤ MUAC < 125 mm) corresponding to moderate acute malnutrition; and MUAC ≥ 125 mm corresponding to the absence of acute malnutrition. The different models are shown in *Table 2*. The height-only model and the three “sub-models” (i.e. height model stratified by three MUAC classes) were each adapted to yield narrow weight classes by solving the appropriate estimation formula for whole kg weights between 2 kg and 25 kg.

**Table 2:** Weight estimation models (n = 1,800,322).

Model	Sub-model	mean	*α*_*1*_	*β*_*1*_	*β*_*2*_	Notes
**MUAC*x***	NA	11.14001	-8.9244	0.1412	0.4212	MUAC-only model (“MUAC1”)
**HEIGHT*x***	NA	11.16397	-7.9416	0.2238	0.8595	Height-only model (“HEIGHT1”). This model was adapted to yield narrow weight classes by solving the appropriate estimation formula for whole kg weights between 2 kg and 25 kg. This model (“HEIGHT2”) could be used to produce a banded weight estimation tape based on height.
	MUAC < 115 mm	7.105647	-5.9172	0.1790	0.7791	Height-based models for different MUAC classes. The three “sub-models” (i.e. the height model stratified by three MUAC classes) were each adapted to yield narrow weight classes by solving the appropriate estimation formula for whole kg weights between 2 kg and 25 kg. These models are “HEIGHT3” when combined and could be used to produce a banded weight estimation tape based on height and MUAC.
	115 mm ≤ MUAC < 125 mm	8.116321	-5.4309	0.1789	0.8553
	MUAC ≥ 125 mm	11.46866	-7.3576	0.2179	0.8529

## Results

Characteristics of the study population are presented in *Table 1*. Summary statistics (i.e. MPD, PW10, PW20, weighted Kappa, and Bland-Altman bias and 95% LOA) are compared in *Table 3* for the height-only BT (2007 [B] and 2011 [A]), MUAC-only (MUAC [HK] and database-derived MUAC1), and database-derived height-only (HEIGHT1) weight estimation methods. Except for the HEIGHT1 model (which is accurate and precise), one-dimensional weight estimation methods (i.e. both BT and both MUAC models) generally performed poorly: BT 2011 [A] has worse accuracy and precision compared to BT 2007 [B]; both BT 2011 [A] and BT 2007 [B] overestimate weight significantly in children with moderate to severe acute malnutrition, less so in children with moderate to severe stunting; MUAC (HK) has poor accuracy and precision; and MUAC1 has good accuracy but poor precision.

**Table 3:** Comparison of Broselow Tape, Hong Kong formula, MUAC-only (MUAC1), and height-only (HEIGHT1) weight estimation models (n = 1,800,322).

				MPD (SD)*				
Variable	Class		N (%)	BT 2007 [B]	BT 2011 [A]	MUAC (HK)	MUAC1	HEIGHT1
**Weight** **(kg)**	(0,25]	weight ≤ 25 kg	1,800,322 (100)	- 4.74 (9.98)	- 9.00 (10.74)	- 13.64 (27.28)	+ 0.48 (29.73)	+ 0.45 (10.05)
	(0,10]	weight ≤ 10 kg	677,164 (37.6)	- 9.05 (11.02)	- 12.54 (11.33)	- 20.23 (34.13)	+ 1.69 (38.62)	+ 1.63 (10.98)
	(10,25]	10 kg < weight ≤ 25 kg	1,123,158 (62.4)	- 2.37 (8.99)	- 6.96 (10.08)	- 10.28 (23.69)	- 0.05 (25.84)	- 0.22 (9.54)
**MUAC (mm)**	< 115	severe wasting	37,294 (2.1)	- 20.76 (12.66)	- 25.36 (12.93)	+61.45 (21.01)	+98.12 (19.08)	- 8.34 (12.20)
	115 ≤ MUAC < 125	moderate wasting	122,677 (6.8)	- 15.16 (10.79)	- 19.24 (10.64)	+21.90 (18.28)	+50.59 (15.38)	- 4.73 (11.16)
	≥ 125	normal	1,640,351 (91.1)	- 3.20 (9.58)	- 8.02 (10.25)	- 17.16 (35.36)	- 3.89 (26.89)	+ 0.98 (9.79)
**WHZ**	WHZ < -3	severe wasting	46,803 (2.6)	- 30.91 (7.65)	- 36.45 (7.14)	+19.02 (39.63)	+47.12 (47.71)	- 22.34 (9.35)
	-3 ≤ WHZ < -2	moderate wasting	164,965 (9.2)	- 20.12 (5.28)	- 25.33 (5.29)	+ 5.76 (26.96)	+27.08 (29.67)	- 13.64 (6.61)
	WHZ ≥ -2	normal	1,588,554 (88.2)	- 2.83 (8.92)	- 6.97 (9.34)	- 16.16 (26.01)	- 2.88 (27.94)	+ 2.17 (9.08)
**HAZ**	HAZ < -3	severe stunting	272,621 (15.1)	- 5.07 (11.36)	- 8.49 (12.02)	- 12.14 (29.54)	+ 6.11 (34.73)	+ 2.80 (9.96)
	-3 ≤ HAZ < -2	moderate stunting	380,993 (21.2)	- 4.40 (10.30)	- 8.07 (10.47)	- 15.45 (16.21)	- 0.19 (29.82)	+ 1.47 (9.56)
	HAZ ≥ -2	normal	1,146,708 (63.7)	- 4.79 (9.69)	- 9.42 (10.66)	- 13.34 (27.01)	- 0.45 (28.68)	- 0.45 (10.12)
**WAZ**	WAZ < -3	severe underweight	134,491 (7.5)	- 18.46 (10.63)	- 22.45 (10.76)	+ 6.29 (33.46)	+32.45 (39.54)	- 8.55 (10.22)
	-3 ≤ WAZ < -2	moderate underweight	328,643 (18.3)	- 11.50 (8.67)	- 15.46 (9.33)	- 7.92 (26.75)	+10.89 (29.56)	- 4.63 (9.56)
	WAZ ≥ -2	normal	1,337,188 (74.3)	- 1.96 (8.96)	- 6.24 (9.35)	- 16.69 (26.48)	- 4.45 (27.76)	+ 2.50 (9.45)
			PW20** % **(95% CI)**	91.19 (91.15; 91.23)	84.00 (83.94; 84.05)	49.48 (49.41; 49.56)	49.85 (49.77; 49.92)	93.98 (93.95; 94.02)
			PW10** % **(95% CI)**	63.33 (63.26; 63.40)	51.64 (51.57; 51.71)	26.34 (26.28; 26.41)	26.66 (26.60; 26.73)	67.44 (67.37; 67.51)
			**Kappa^ (95% CI)**	0.8794 (0.8790; 0.8797)	0.8708 (0.8705; 0.8711)	0.5619 (0.5610; 0.5630)	0.5760 (0.5751; 0.5770)	0.8832 (0.8829; 0.8835)
			**Bland Altman bias^^ (kg)**	- 0.46	- 0.94	- 1.43	+0.05	+0.04
			**Bland Altman 95% LOA^^ (kg)**	- 2.50; +1.57	- 3.26; +1.39	- 7.25; +4.38	- 6.37; +6.46	- 2.08; +2.17

Severe wasting = severe acute malnutrition; moderate wasting = moderate acute malnutrition; WHZ = weight-for-height z-score; HAZ = height-for-age z-score; WAZ = weight-for-age z-score

*Percentage difference is calculated as:
$$percentage\;difference=\frac{true\;weight-estimated\;weight}{true\;weight}\;\times \;100$$

Mean percentage difference (MPD) and standard deviation (SD) percentage difference were estimated using Huber M estimators of location and scale [14]. The mean percentage difference is a measure of systematic bias or accuracy (i.e. lower MPD = better accuracy). Positive MPD values indicate underestimation of true weight. Negative MPD values indicate overestimation of true weight. The SD percentage difference is a measure of precision (i.e. lower SD = better precision). The difference in accuracy between any pair of methods can be assessed using the ratio of the absolute values of their mean percentage difference. The difference in precision between any pair of methods can be assessed using the ratio of their SD percentage differences. For example, (in Table 3) comparing BT 2011 (A) and HEIGHT 1 in all children:

$\Delta \;accuracy=\frac{\left|-9.00\right|}{\left|0.45\right|}=20.00\;\times \;improvement;\;\Delta \;precision=\frac{10.74}{10.05}=1.07\;\times \;improvement$

Values above one indicate better performance. Values of one indicate no difference in performance. Values below one indicate worse performance.

**PW10/PW20: are the proportion of estimates accurate to within ±10%/±20% of true weight and are expressed as a point estimate and 95% confidence interval. PW10 and PW20 are measures of accuracy (i.e. higher proportion = better accuracy).

**^Kappa:** Weighted Kappa statistic is a measure of inter-rater agreement for qualitative (categorical) items and is expressed as a point estimate and 95% confidence interval. The weighted Kappa is a measure of accuracy (higher Kappa = better accuracy).

**^^Bland-Altman bias (95% LOA):** Bland-Altman bias (mean of true–estimated weight, or mean error) and Bland-Altman 95% limits of agreement (mean difference [1.96 SD]) were calculated following the method of Bland & Altman [15]. The Bland-Altman bias is a measure of accuracy (lower bias = better accuracy). The Bland-Altman 95% LOA provide a measure of precision (narrower LOA = better precision).

Results of weight estimation by HEIGHT1 fitted separately for three MUAC classes are presented in *Table 4*. For MUAC < 115 mm, PW10/PW20 were 63.91% (95% CI 63.42%, 64.40%)/90.72% (95% CI 90.42%, 91.01%); and bias (LOA) were +0.14 kg (-1.29 kg; +1.56 kg). For 115 mm ≤ MUAC < 125 mm, PW10/PW20 were 76.27% (95% CI 76.03%, 76.51%)/96.36% (95% CI 96.25%, 96.46%); and bias (LOA) were +0.06 kg (-1.20 kg; +1.33 kg). For MUAC > 125 mm, PW10/PW20 were 69.93% (95% CI 69.86%, 70.00%)/95.27% (95% CI 95.24%, 95.30%); and bias (LOA) were +0.05 kg (-2.04 kg; +2.13 kg).

**Table 4:** Weight estimation by height-only model (HEIGHT1) fitted for three MUAC classes (n = 1,800,322).

		MPD (SD)		
Variable	Class	MUAC < 115 mm	115 mm ≤ MUAC < 125 mm	MUAC ≥ 125 mm
**Weight** **(kg)**	(0,25]	+ 2.08 (10.69) n = 37,294	+ 0.86 (8.25) n = 122,677	+ 0.49 (9.55) n = 1,640,351
	(0,10]	+ 1.84 (10.21) n = 33,612	+ 0.77 (8.09) n = 103,617	+ 1.99 (10.10) n = 539,935
	(10,25]	+ 5.18 (17.67) n = 3,682	+ 1.66 (10.40) n = 19,060	- 0.21 (9.27) n = 1,100,416
**MUAC** **(mm)**	< 115	+ 2.08 (10.69) n = 37,294	NA	NA
	115 ≤ MUAC < 125	NA	+ 0.86 (8.25) n = 122,677	NA
	≥ 125	NA	NA	+ 0.49 (9.55) n = 1,640,351
**WHZ**	WHZ < -3	- 6.98 (8.11) n = 13,324	- 12.26 (5.10) n = 13,115	- 26.56 (5.83) n = 20,364
	-3 ≤ WHZ < -2	+2.16 (6.78) n = 12,573	- 4.08 (4.20) n = 39,916	- 15.74 (4.54) n = 112,476
	WHZ ≥ -2	+13.25 (8.54) n = 11,397	+ 5.99 (5.97) n = 69,646	+ 1.71 (8.77) n = 1,507,511
**HAZ**	HAZ < -3	+2.84 (10.20) n = 15,464	+ 2.06 (7.83) n = 35,937	+ 3.41 (9.14) n = 221,220
	-3 ≤ HAZ < -2	+1.95 (10.39) n = 8,480	+ 1.03 (8.16) n = 30,007	+ 1.52 (9.05) n = 342,506
	HAZ ≥ -2	+1.23 (11.56) n = 13,350	- 0.02 (8.59) n = 56,733	- 0.45 (9.68) n = 1,076,625
**WAZ**	WAZ < -3	- 1.01 (9.58) n = 22,330	- 3.27 (7.34) n = 38,827	- 8.39 (9.53) n = 73,334
	-3 ≤ WAZ < -2	+3.54 (9.37) n = 8,494	+ 0.24 (7.31) n = 44,924	- 5.13 (9.02) n = 275,225
	WAZ ≥ -2	+12.55 (12.26) n = 6,470	+ 6.10 (8.39) n = 38,926	+ 2.13 (9.05) n = 1,291,792
**PW20% (95% CI)**		90.72 (90.42; 91.01)	96.36 (96.25; 96.46)	95.27 (95.24; 95.30)
**PW10% (95% CI)**		63.91 (63.42; 64.40)	76.27 (76.03; 76.51)	69.93 (69.86; 70.00)
**Kappa (95% CI)**		0.8255 (0.8215; 0.8295)	0.8430 (0.8412; 0.8451)	0.8754 (0.8750; 0.8757)
**Bland Altman bias (kg)**		+0.14	+0.06	+0.05
**Bland Altman 95% LOA (kg)**		- 1.29; +1.56	- 1.20; +1.33	- 2.04; +2.13

WHZ = weight-for-height z-score; HAZ = height-for-age z-score; WAZ = weight-for-age z-score

**Fig 1:**
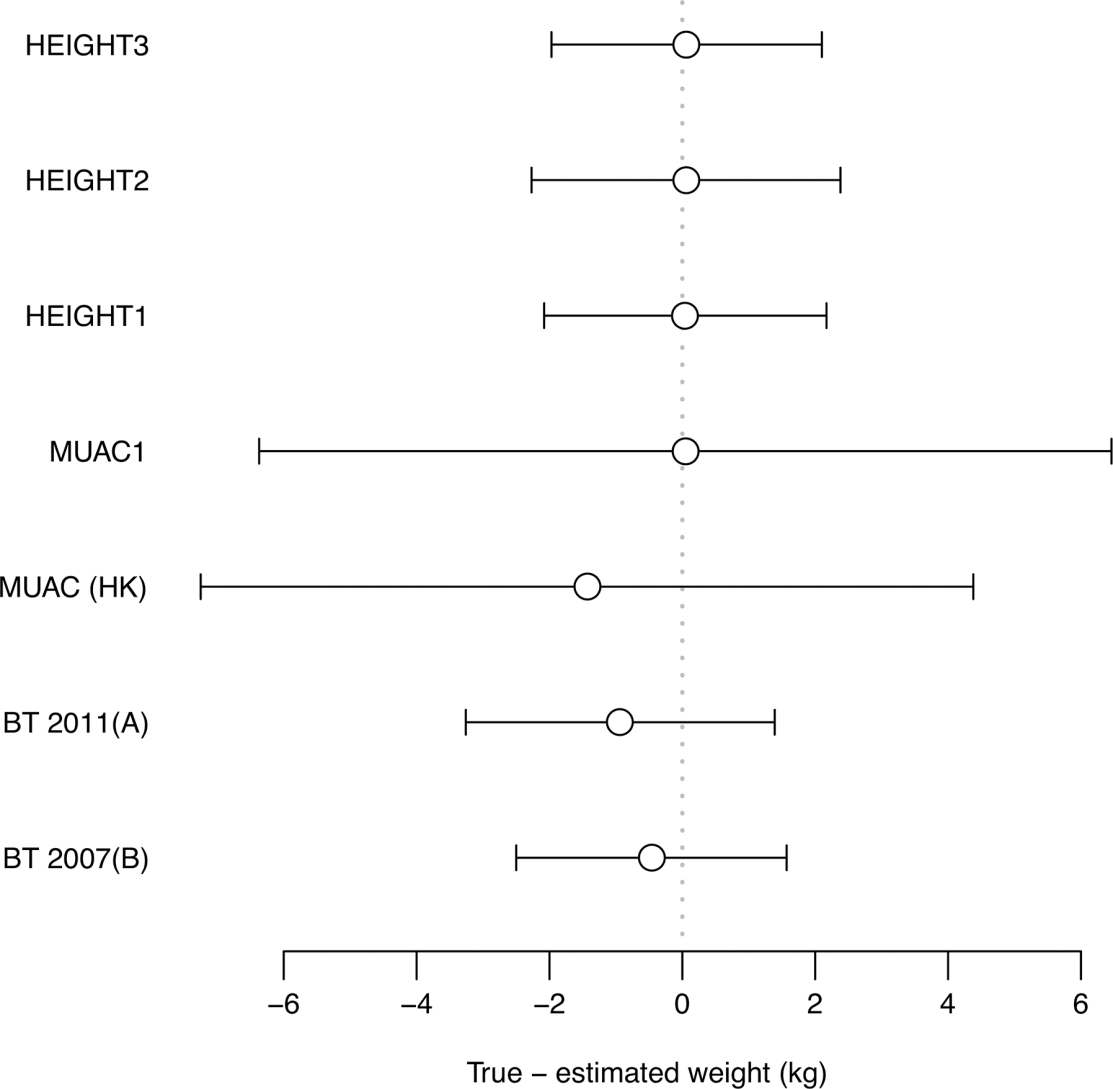
Forest plot showing Bland-Altman bias and 95% limits of agreement for weight estimation models. Bias (circles) is a measure of accuracy (lower absolute values = better accuracy). 9%% LOA (error bars) is a measure of precision (narrower bars = better precision).

Results of weight estimation for the one-dimensional height-only (HEIGHT2) and two-dimensional height + MUAC (HEIGHT3) models are presented in *Table 5.* Both HEIGHT2 and HEIGHT3 models are accurate and precise. For HEIGHT3, MPD was +0.67% (SD = 9.95%); PW10/PW20 were 68.31% (95% CI 68.24%, 68.38%)/94.73% (95% CI 94.69%, 94.76%); and bias (LOA) were +0.06 kg (-1.97 kg; +2.10 kg). The HEIGHT3 model is superior to the HEIGHT2 model in terms of accuracy: 95% confidence limits for HEIGHT3 compared to HEIGHT2 are 68.24; 68.38 and 65.65; 65.79 for PW10, 94.69; 94.76 and 93.36; 93.43 for PW20, and 0.8727; 0.8733 and 0.8674; 0.8681 for weighted Kappa. A comparison of Bland-Altman bias and 95% LOA for HEIGHT2 and HEIGHT3 models is presented in *Fig 1*. Comparison of HEIGHT3 subgroups by survey period and WHO Region are presented in *Table 6*. The model performed similarly with data from different time periods (i.e. 1992–2006 inclusive and 2007–2017 inclusive) and WHO Regions (*Table 6*).

**Table 5:** Weight estimation by height-only (HEIGHT 2) and height + MUAC (HEIGHT 3) models (n = 1,800,322).

		MPD (SD)	
Variable	Class	HEIGHT2	HEIGHT3
**Weight** **(kg)**	(0,25]	+ 0.59 (10.57)	+ 0.67 (9.95)
	(0,10]	+ 1.86 (11.15)	+ 1.92 (10.32)
	(10,25]	- 0.11 (9.88)	- 0.04 (9.70)
**MUAC** **(mm)**	< 115	- 7.96 (13.01)	+ 2.42 (11.92)
	115 ≤ MUAC < 125	- 4.48 (11.89)	+ 1.05 (9.57)
	≥ 125	+ 1.10 (10.20)	+ 0.61 (9.97)
**WHZ**	WHZ < -3	- 22.24 (10.07)	- 16.95 (12.36)
	-3 ≤ WHZ < -2	- 13.52 (7.28)	- 11.44 (8.80)
	WHZ ≥ -2	+ 2.32 (9.42)	+ 2.13 (9.08)
**HAZ**	HAZ < -3	+ 3.01 (10.56)	+ 3.36 (9.89)
	-3 ≤ HAZ < -2	+ 1.64 (10.02)	+ 1.63 (9.50)
	HAZ ≥ -2	- 0.33 (10.59)	- 0.30 (10.05)
**WAZ**	WAZ < -3	- 8.43 (10.79)	- 5.56 (10.23)
	-3 ≤ WAZ < -2	- 4.48 (9.83)	- 4.07 (9.55)
	WAZ ≥ -2	+ 2.62 (9.63)	+ 2.40 (9.79)
**PW20% (95% CI)**		93.40 (93.36; 93.43)	94.73 (94.69; 94.76)
**PW10% (95% CI)**		65.72 (65.65; 65.79)	68.31 (68.24; 68.38)
**Kappa (95% CI)**		0.8677 (0.8674; 0.8681)	0.8730 (0.8727; 0.8733)
**Bland Altman bias (kg)**		+0.06	+0.06
**Bland Altman 95% LOA (kg)**		- 2.27; +2.38	- 1.97; +2.10

WHZ = weight-for-height z-score; HAZ = height-for-age z-score; WAZ = weight-for-age z-score

**Table 6:** Comparison of height + MUAC model (HEIGHT3) subgroups by survey period and WHO Region.

Survey Period	1992–2006	2007–2017	1992–2017					
WHO Region*	All	All	Africa	Americas	South-East Asia	Europe	Eastern Mediterranean	Western Pacific
**Countries n**	32	51	32	2	7	2	7	1
**Children n**	535,241	1,265,081	1,280,439	40,073	48,968	5,189	419,558	6,095
**MPD (SD)**	+0.81 (9.89)	+0.61 (10.08)	+1.12 (9.63)	+3.71 (8.62)	- 1.63 (8.96)	+5.02 (8.74)	- 0.79 (10.26)	+0.37 (8.59)
**PW20** **%** **(95% CI)**	95.13 (95.07; 95.19)	94.56 (94.52; 94.60)	95.02 (94.98; 95.06)	95.79 (95.58; 95.98)	96.35 (96.18; 96.51)	93.39 (92.67; 94.04)	93.54 (93.46; 93.61)	95.93 (95.40; 96.41)
**PW10** **%** **(95% CI)**	68.90 (68.77; 69.02)	68.06 (67.98; 68.14)	68.70 (8.62; 68.78)	70.07 (69.62; 70.52)	72.08 (71.68; 72.47)	64.21 (62.89; 65.52)	66.47 (66.33; 66.61)	74.78 (73.67; 75.87)
**Kappa** **(95% CI)**	0.8797 (0.8792;0.8803)	0.8699 (0.8695;0.8703)	0.8717 (0.8713;0.8721)	0.8475 (0.8451;0.8498)	0.8871 (0.8853;0.8889)	0.8529 (0.8463;0.8594)	0.8773 (0.8766; 0.8779)	0.8755 (0.8700;0.8810)
**Bland Altman** **bias (kg)**	+0.08	+0.06	+0.12	+0.41	- 0.19	+0.58	- 0.11	+0.04
**Bland Altman** **95% LOA (kg)**	- 1.95; +2.12	-1.98; +2.09	- 1.92; +2.15	- 1.62; +2.45	- 1.93; +1.56	- 1.46; +2.61	- 2.14; +1.93	- 1.70; +1.79

*Countries Represented in Surveys by WHO Region (16): Africa: Angola; Benin; Burkino Faso; Burundi; Cameroon; Central African Republic; Chad; Democratic Republic of the Congo (Kinshasa); Côte d’Ivoire; Eritrea; Ethiopia; Gambia; Guinea; Guinea-Bissau; Kenya; Liberia; Madagascar; Malawi; Mali; Mauritania; Mozambique; Niger; Nigeria; Rwanda; Senegal; Sierra Leone; South Sudan; Togo; Uganda; United Republic of Tanzania; Zambia; Zimbabwe; **Americas:** Guatemala; Haiti; **South-East Asia:** Bangladesh; India; Indonesia; Myanmar; Nepal; Sri Lanka; Thailand; **Europe:** Albania; Tajikistan; **Eastern Mediterranean:** Afghanistan; Djibouti; Jordan; Pakistan; Somalia; Sudan; Yemen; **Western Pacific:** Philippines.

**Fig 2:**
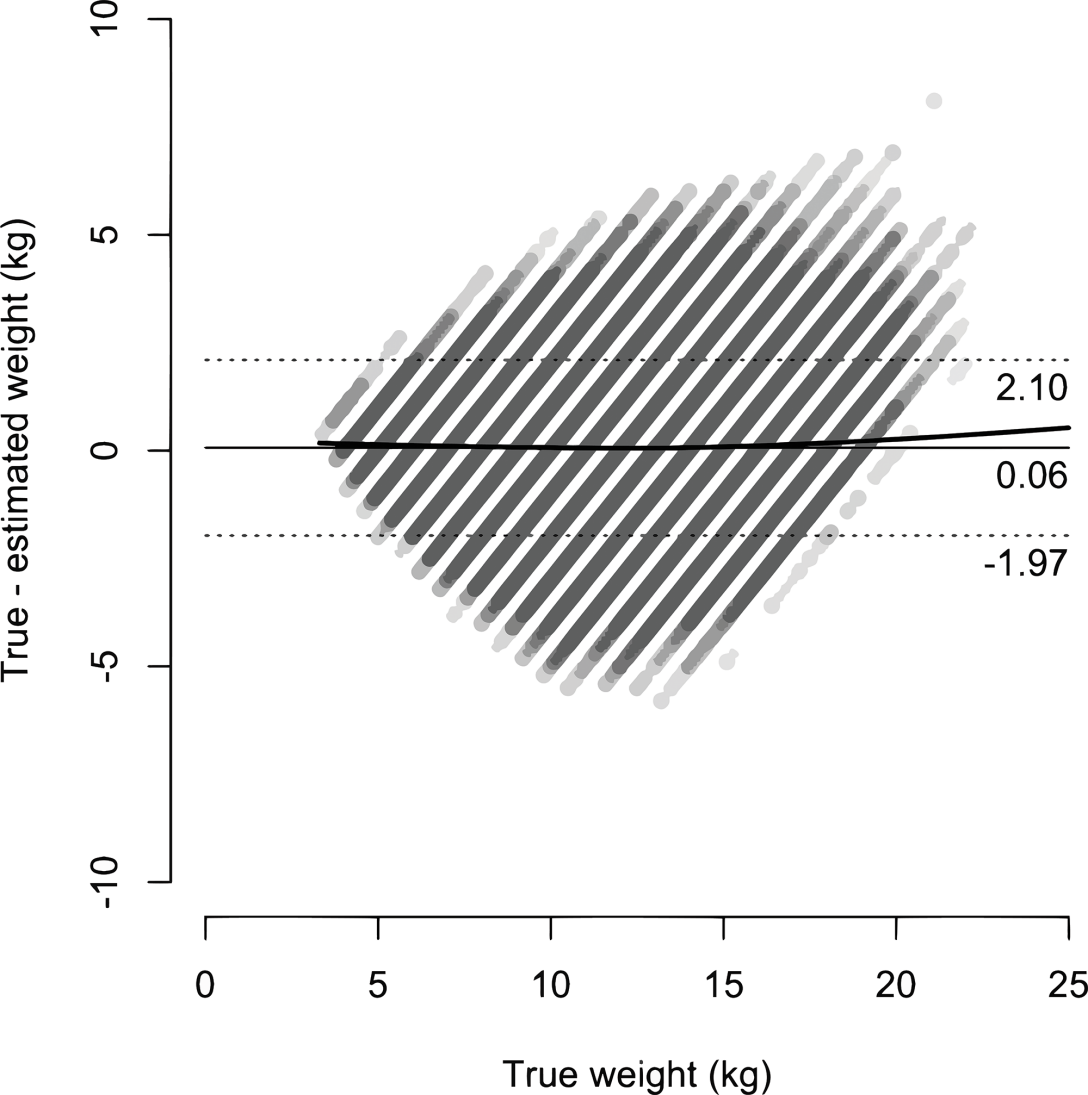
Bland-Altman plot of height + MUAC (HEIGHT3) model. Bias (dark line); 95% LOA (dotted lines).

**Fig 3:**
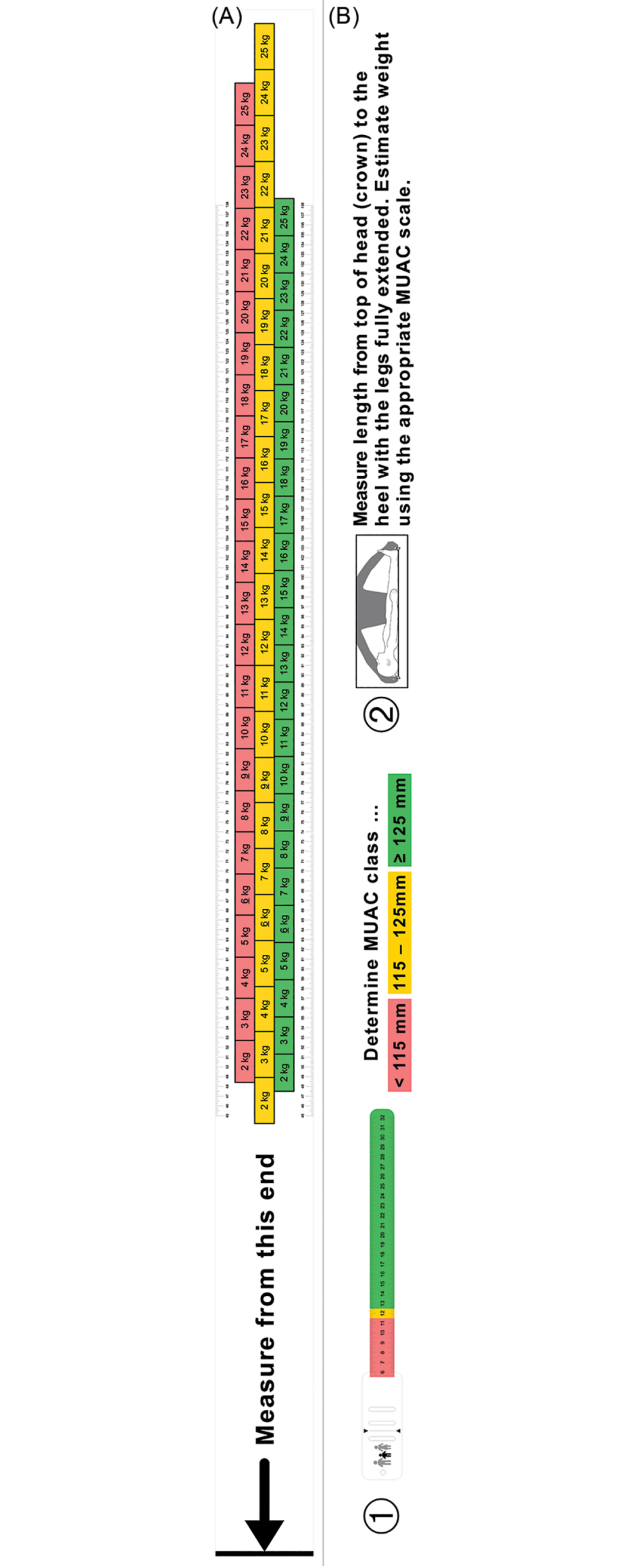
Proposed weight estimation tape based on height + MUAC (HEIGHT3) model. (A) Scale drawing of height-based tape stratified according to 3 MUAC classes. (B) Step 1. Determine MUAC class with MUAC tape (red = MUAC < 115 mm; yellow = 115mm ≤ MUAC < 125 mm; green = MUAC ≥ 125 mm). Step 2. Measure length (in cm) from top of head (crown) to the heel with legs full extended. Estimate weight (in kg) using the appropriate MUAC scale.

Narrow weight classes based on HEIGHT2 and HEIGHT3 models are presented in *Table 7*. A Bland-Altman plot of the HEIGHT3 model is presented in *Fig 2*. An annotated scale drawing of a proposed weight estimation tape is presented in *Fig 3*.

**Table 7:** Weight class by height-only (HEIGHT2) and height + MUAC (HEIGHT3) models.

Weight Class (kg)	HEIGHT2		HEIGHT3					
			MUAC < 115 mm		115 mm ≤ MUAC < 125 mm		MUAC ≥ 125 mm	
	Height Low (cm)	Height High (cm)	Height Low (cm)	Height High (cm)	Height Low (cm)	Height High (cm)	Height Low (cm)	Height High (cm)
**2**	48.4	52.1	48.4	52.7	44.2	48.9	47.5	51.3
**3**	52.2	55.9	52.8	57.1	49.0	53.7	51.4	55.2
**4**	56.0	59.8	57.2	61.4	53.8	58.4	55.3	59.1
**5**	59.9	63.6	61.5	65.8	58.5	63.2	59.2	63.0
**6**	63.7	67.5	65.9	70.1	63.3	68.0	63.1	66.9
**7**	67.6	71.3	70.2	74.5	68.1	72.8	67.0	70.9
**8**	71.4	75.1	74.6	78.8	72.9	77.6	71.0	74.8
**9**	75.2	79.0	78.9	83.2	77.7	82.3	74.9	78.7
**10**	79.1	82.8	83.3	87.5	82.4	87.1	78.8	82.6
**11**	82.9	86.7	87.6	91.9	87.2	91.9	82.7	86.5
**12**	86.8	90.5	92.0	96.2	92.0	96.7	86.6	90.4
**13**	90.6	94.3	96.3	100.6	96.8	101.5	90.5	94.3
**14**	94.4	98.2	100.7	104.9	101.6	106.3	94.4	98.2
**15**	98.3	102.0	105.0	109.3	106.4	111.0	98.3	102.2
**16**	102.1	105.9	109.4	113.6	111.1	115.8	102.3	106.1
**17**	106.0	109.7	113.7	118.0	115.9	120.6	106.2	110.0
**18**	109.8	113.6	118.1	122.3	120.7	125.4	110.1	113.9
**19**	113.7	117.4	122.4	126.7	125.5	130.2	114.0	117.8
**20**	117.5	121.2	126.8	131.0	130.3	134.9	117.9	121.7
**21**	121.3	125.1	131.1	135.4	135.0	139.7	121.8	125.6
**22**	125.2	128.9	135.5	139.7	139.8	144.5	125.7	129.6
**23**	129.0	132.8	139.8	144.1	144.6	149.3	129.7	133.5
**24**	132.9	136.6	144.2	148.4	149.4	154.1	133.6	137.4
**25**	136.7	140.4	148.5	152.8	154.2	158.9	137.5	141.3

**WEIGHT ESTIMATION EXAMPLE:** If height is 56 cm and MUAC is < 115 mm, estimated weight is 3 kg; if height is 56 cm and MUAC is > 125 mm, estimated weight is 4 kg. **Caution:** Extrapolation should be limited to (at most) 15% below (i.e. 44.5 cm) and 15% above (i.e. 138 cm) database height limits shown in *Table 1*.

## Discussion

This study confirmed the accuracy and precision of a weight estimation tool developed from a nutritional survey database of 453,990 children aged 6 to 59 months of age in 32 low-to-middle income countries during 1992–2006 with a database of 1,800,322 children in 51 low-to-middle income countries during 1992–2017 [5, 6]. The accuracy and precision of the tool did not vary significantly by survey period (i.e. 1992–2006 inclusive and 2007–2017 inclusive) or WHO Region [16]. The tool was tested in locations with high prevalence of acute and chronic malnutrition. In these limited-resource settings, a scale to measure weight may not be immediately available to healthcare professionals including first-response providers. The two-dimensional tool (i.e. based on height stratified by three MUAC classes) had been found to be more accurate and precise than existing one-dimensional weight estimation methods (i.e. based on either height or MUAC alone) [6]. This finding was confirmed by this study. Two-dimensional weight estimation methods have been found to be more accurate than one-dimensional methods in estimating total body weight [4]. As severe acute malnutrition is a significant contributor to under-five mortality, a tool which predicts total body weight (i.e. vs ideal body weight) would be preferable in low-to-middle income countries where nearly all global under-five mortality occurs because it avoids overestimation of weight in the undernourished child [3].

Ideal characteristics of a tool used for weight estimation, especially when needed urgently during resuscitation, are simplicity, accuracy, and precision. Simplicity is the consequence of the complexity of the required measurement / estimate and the design of a measurement / estimation tool. Accuracy of a measurement / estimation tool is the degree of nearness of a measurement / estimate to the true value. In this study, MPD, PW10, PW20, weighted Kappa statistic, and Bland-Altman bias (i.e. the mean of true–estimated weight) were used as measures of tool accuracy [15, 17–19]. Precision of a measurement / estimation tool is the degree of reproducibility of repeated measurements / estimates. In this study, MPD SD and Bland-Altman 95% LOA were used as measures of tool precision [15].

The tool was developed first as a linear model to estimate weight directly from measured height in the international database. After the initial linear model was corrected to reduce error, the linear-rotated, height-only model HEIGHT1 was found to be: more accurate than either BT or MUAC alone; more precise than MUAC alone; and of similar precision to BT. A simple linear tape based on HEIGHT1 was considered for field use to estimate weight in children. However, the accuracy of HEIGHT1 model as a weight estimation tool was noted to deteriorate according to severity of malnutrition measured by MUAC, weight-for-height z-score, height-for-age z-score, and weight-for-age z-score (*Table 3*). The HEIGHT1 model was then fitted for three separate MUAC classes (i.e. severe acute malnutrition, moderate acute malnutrition, absence of malnutrition); this procedure yielded improved accuracy and precision compared to HEIGHT1 (*Table 4*). The fitted model was then converted to two additional models based on sequential 1 kg wide weight classes (i.e. HEIGHT2 for height only and HEIGHT3 for height and MUAC class) without loss of accuracy or precision (*Table 5*).

The weight classes which are shown in *Table 7* could be used to produce a height-to-weight tape with stratification according to nutritional status defined by MUAC class. The weight classes in *Table 7* reflect the more extensive data sampling of this study without change in accuracy or precision compared to the previous study and therefore replace those in Table 4 of the previous study [6].

The study had potential limitations. Firstly, testing of BT was virtual. It is unclear if this would lead to significantly different outcomes of accuracy and precision compared to live testing. Secondly, the results of this study are applicable to children in low-to-middle income countries with age between 6 and 59 months, weight between 3.3 and 25 kg, length / height between 44.5 and 138 cm, and MUAC between 80 and 179 mm (*Table 1* and *Table 7*). Thirdly, we were unable to directly compare our methodology to other two-dimensional methods used for weight estimation in children (e.g. Mercy and PAWPER) because requisite data (i.e. humeral length for Mercy; visual appraisal of body habitus for PAWPER) were not obtained in the nutritional anthropometry surveys reported in our study [4, 5].

## Conclusions

A model which estimated weight directly from database height and MUAC in children in low-to-middle income countries with high prevalence of acute and chronic malnutrition was confirmed to be accurate and precise. A simple height-based weight estimation tape stratified according to MUAC is proposed for children aged 6–59 months in limited-resource settings.
