# Correction: Weight estimation for children aged 6 to 59 months in limited-resource settings: A proposal for a tape using height and mid-upper arm circumference

**DOI:** 10.1371/journal.pone.0202783

**Published:** 2018-08-16

**Authors:** Mark E. Ralston, Mark A. Myatt

There is an error in the caption for Fig 1, “Forest plot showing Bland-Altman bias and 95% limits of agreement for weight estimation models.” Please see the complete, correct Fig 1 caption here.

**Fig 1:**
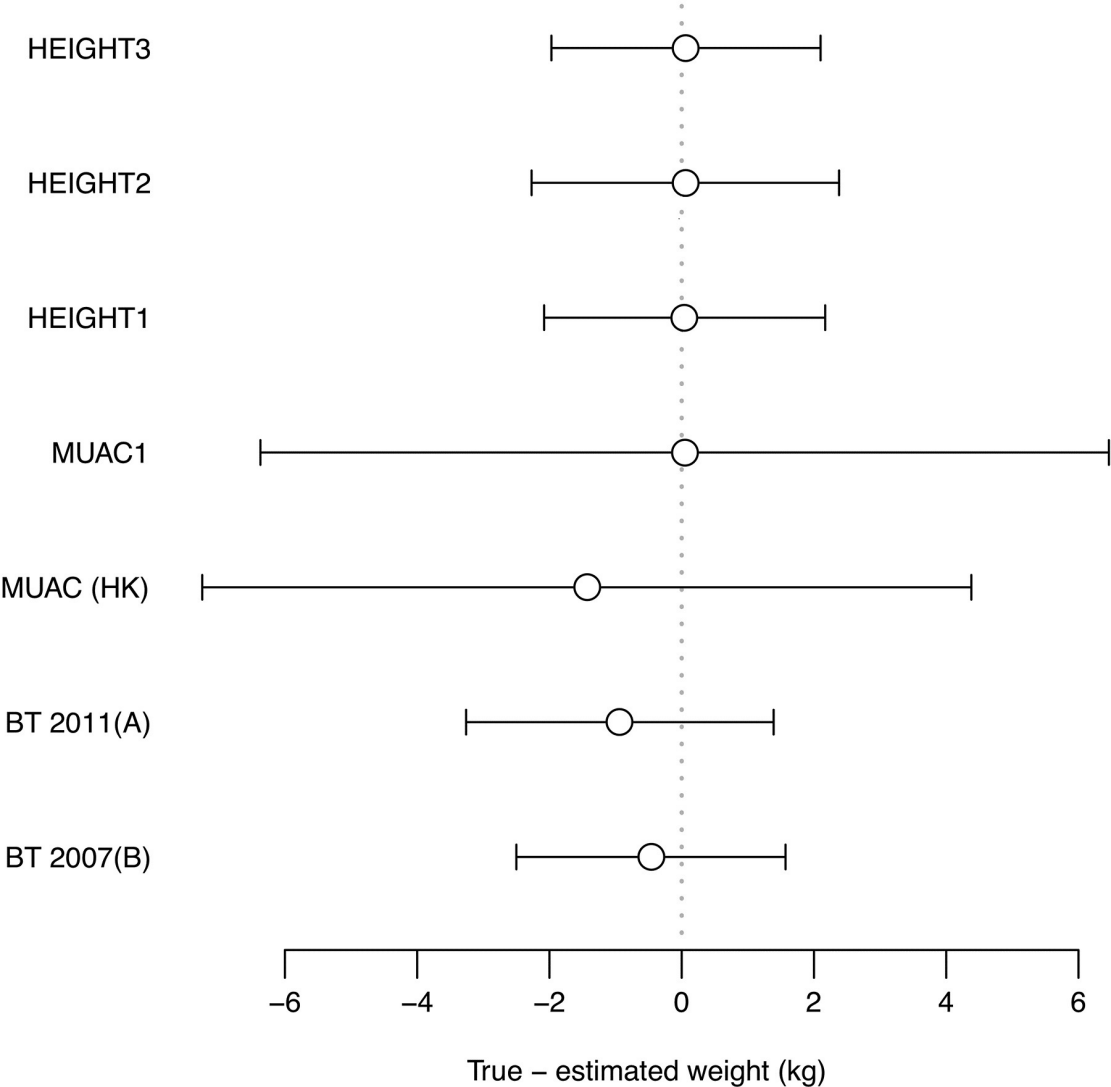
Forest plot showing Bland-Altman bias and 95% limits of agreement for weight estimation models. Bias (circles) is a measure of accuracy (lower absolute values = better accuracy). 95% LOA (error bars) is a measure of precision (narrower bars = better precision).
